# The Role of the NLRP3 Inflammasome in HCC Carcinogenesis and Treatment: Harnessing Innate Immunity

**DOI:** 10.3390/cancers14133150

**Published:** 2022-06-27

**Authors:** Stavros P. Papadakos, Nikolaos Dedes, Elias Kouroumalis, Stamatios Theocharis

**Affiliations:** 1 First Department of Pathology, Medical School, National and Kapodistrian University of Athens, 11527 Athens, Greece; stavrospapadakos@gmail.com (S.P.P.); ndedes@med.uoa.gr (N.D.); 2 Department of Gastroenterology, Medical School, University of Crete, 71500 Heraklion, Greece; kouroum@med.uoc.gr

**Keywords:** HCC, NLRP3, innate immunity, inflammasome

## Abstract

**Simple Summary:**

The Hepatocellular Carcinoma (HCC) remains a major concern for the public health. The pandemic of metabolic syndrome in the Western societies and the considerable amounts of viral hepatitis in the underdeveloped countries keep fueling s the development of HCC. The hepatotropic viruses evade the NLRP3 inflammasome in order to sustain the chronicity of infection leading to cirrhosis while in the established tumors the activation of NLRP3 promotes several pro-tumorigenic effects. That leads to substantial economic burden for the societies and alternative therapeutic targets should be investigated. Reviewing past and more recent literature it can be deduced that the NLRP3 inflammasome could be an ideal therapeutic effects and it should be studied in more depth.

**Abstract:**

The HCC constitutes one of the most frequent cancers, with a non-decreasing trend in disease mortality despite advances in systemic therapy and surgery. This trend is fueled by the rise of an obesity wave which is prominent the Western populations and has reshaped the etiologic landscape of HCC. Interest in the nucleotide-binding domain leucine-rich repeat containing (NLR) family member NLRP3 has recently been revived since it would appear that, by generating inflammasomes, it participates in several physiologic processes and its dysfunction leads to disease. The NLRP3 inflammasome has been studied in depth, and its influence in HCC pathogenesis has been extensively documented during the past quinquennial. Since inflammation comprises a major regulator of carcinogenesis, it is of paramount importance an attempt to evaluate the contribution of the NLRP3 inflammasome to the generation and management of HCC. The aim of this review was to examine the literature in order to determine the impact of the NLRP3 inflammasome on, and present a hypothesis about its input in, HCC.

## 1. Introduction

### 1.1. Epidemiology of Hepatocellular Carcinoma

Hepatocellular carcinoma (HCC) comprises the most common primary liver cancer. Globally, it ranks fifth in terms of incidence and second in terms of cancer-related mortality, indicating that it will be at the forefront of public health awareness in the coming decades [1]. HCC arises in the vast majority of cases as the end-result of chronic infection with viral hepatitis B and C (HBV and HCV), alcohol abuse and in the context of non-alcoholic fatty liver disease (NAFLD)/non-alcoholic steatohepatitis (NASH) [2]. The latter is the hepatic manifestation of obesity and metabolic syndrome. Progress towards the augmentation of NAFLD-driven HCC diagnoses has been achieved [3] in recent decades. In fact, NAFLD-induced HCC is expected to become the leading indication of liver transplantation in the years to come [4]. The development of cirrhosis substantially enhances the risk of HCC development [5]. In terms of molecular classification, two main categories have been recognized: a proliferative group and a non-proliferative one. The first constitutes a more aggressive disease spectrum, with poor cellular differentiation, elevated alpha-fetoprotein (AFP) levels, the presence of *TP53* and chromosomal instability. It is associated with hepatitis B (HBV) infection and a poor clinical outcome. The non-proliferative class, on the other hand, is characterized by a better clinical course as a result of its genetic proximity with normal liver parenchyma. Its primary genetic characteristics are mutations on the *CTNNB1* gene, which encodes the catenin beta-1 protein, an immune-deserted phenotype and a low tumor grade. Clinically, there is an association between HCV infection and chronic alcohol abuse [6,7,8,9]. Inflammation has been recognized as one of the hallmarks of carcinogenesis [10] and multiple immunotherapeutic approaches have been designed to target cancer-specific inflammation. The combination of atezolizumab and bevacizumab has gained ground as a first line therapy over sorafenib, while several immune checkpoint inhibitors (ICIs) are being evaluated in clinical trials [11]. Additionally, PD-L1 and tumor mutational burden (TMB) constitute well-characterized biomarkers that could potentially target those patients that would benefit most from immunotherapy treatments [12,13]. These were ratified by epidemiologic studies which demonstrated a reduced risk of HCC in patients treated with low-dose aspirin [14]. The effects of inflammasomes in various cancers has gained importance in recent years [15,16], with interest in this subject expected to peak soon. Multiple single-nucleotide polymorphisms (SNPs) in inflammation-related genes such as the interleukin (IL)1B gene have been documented to promote the HCC [17]. This could further indicate that inflammasomes might influence carcinogenesis. In fact, references in the literature to the role of the NLRP3 inflammasome in HCC have begun to emerge [18].

### 1.2. Mechanisms of NLRP3 Inflammasome Activation

Inflammasomes are multimeric protein complexes which are a fundamental constituent of the innate immune system. Its responsiveness to stimuli is defined by their sensor molecule, which constitutes a member of the pattern recognition receptors (PRR) system. To date, only five members of the PRR system have been demonstrated to participate in the formation of inflammasomes: the nucleotide-binding oligomerization domain (NOD), NLRP1, NLRP3, NLRC4 and absent-in-melanoma 2 (AIM2) and pyrin [19]. Inflammasomes participate in various physiological processes including the orchestration of the immune response on mucosal surfaces, the shaping of resistance against microorganisms and the mediation of insulin signaling [20]. Their deregulation is linked to a variety of inflammatory [21,22] degenerative and metabolic diseases [23] as well as tumorigenesis [24].

The NLRP3 inflammasome has been identified as a mediator in a wide variety of diseases and as a potential novel therapeutic target. Since its role in the progression of atherosclerosis, heart failure, glomerulonephritis and several infectious diseases [25,26,27,28,29] has been acknowledged, it would be useful to highlight the most fundamental stages of NLRP3 activation. It resides in the cytoplasm and acts as an immune sensor to a plethora of stimuli, producing IL-1b and IL-18 and initiating the immune response. It is arranged in a dodecameric structure by three proteins: an NLRP3 scaffold, an apoptosis-associated speck-like protein (ASC), which functions as adaptor, and caspase-1. An exhaustive presentation of the NLRP3 inflammasome has been conducted elsewhere [30,31] and it goes beyond the scope of this manuscript [32]. The fundamental proposition regarding NLRP3 inflammasome activation constitutes the two-part hypothesis. Concisely, “Signal 1” emerges when pathogen-associated molecular patterns (PAMPs) from microbes and endogenous damage-associated molecular patterns (DAMPs) prime the cell through toll-like receptors (TLRs), the tumor necrosis factor receptor (TNFR) and the IL-1b receptor (IL-1R), activate the nuclear factor kappa B (NF-kB) signaling pathway and upregulate the expression of the NLRP3 oligomers pro-IL-1b and pro- IL-18. “Signal 2” derives mainly from a K+ efflux, which is the end-result of a wide variety of stimuli such as the activation of the P2 × 7 receptor, which is activated in the presence of elevated adenosine triphosphate (ATP) concentrations, the dissolution of lysosomes and the presence of pore-forming toxins. The fact that mitochondrial dysfunction is a source of reactive oxygen species (ROS) and oxidized mitochondrial DNA (mtDNA) also potentiates the assembly of inflammasome monomers into the activated form of the NLRP3 inflammasome. Consequently, the activation of the NLRP3 complex leads to the cleavage of pro-ILs and GasderminD to form pores in the cell membrane, resulting in pyroptosis [32,33].

As is apparent from the above, the stimulants of the NLRP3 inflammasome are extensive, and it is essential to investigate the importance of the NLRP3 inflammasome in the progression of liver disease.

## 2. The NLRP3 Inflammasome in Liver Disease

Our knowledge of the function of the liver has evolved considerably in recent decades. Classically, it was only perceived as the primary metabolism organ, with it regulating the lipid and cholesterol metabolism, the production of albumin and clotting factors, detoxifying the end-products of the metabolism and exerting the glycogen storage. Presently, it is widely recognized as an immune response-orchestrating organ given its role in the generation of acute phase reactants, proteins of complementary systems and anti-microbials. In parallel, the liver maintains an antigen-presenting capacity and can efficiently remove endotoxin [34]. Taking this a step further, due to its unique anatomy, the liver is exposed to a series of portal blood stream-derived signals from the diet (e.g., a high concentration of fats and carbohydrates) and the commensal flora (e.g., PAMPs). The nature of the liver’s immune response is determined by the fact that a delicate balance has to be maintained between the tolerance towards the above mentioned ligands of the PRR system and the elimination of invading pathogens [35]. A synergy of inflammation-inducing and -resolving mechanisms is necessary in order to carry out physiologic functions such as liver regeneration, the resolution of fibrosis, the response to infection and PAMP tolerance, and this synergy is accomplished with the constitutive expression of both pro-inflammatory [IL-2, IL-7, IL-12, IL-15] and interferon-(IFN)γ and anti-inflammatory [IL-10, IL-13 and the transforming growth factor beta(TGFβ)] cytokines [34,36]. The effects of the NLRP3 inflammasome on certain liver diseases are presented below.

### 2.1. The NLRP3 Inflammasome in NAFLD–NASH

The preeminent dysfunction in NASH derives from the accumulation of lipids (such as fatty acids and cholesterol) and the consequent activation of NLRP3 in hepatocytes, immune cells and hepatic stellate cells (HSCs) [37]. Another contributing mechanism comprises the upregulation of NF-κB by a lipopolysaccharide (LPS)-mediated stimulation of the TLR4 [37]. Since, as mentioned above, the NASH epidemic in the Western world is expected to promote NASH to the primary cause of HCC and that a great amount of research has been conducted to elucidate its role in pathogenesis and to target it therapeutically, it would be meaningful to present the relevant data in Table 1.

Such concrete data about the influence of the NLRP3 inflammasome on the molecular pathogenesis of NASH contributed to the concept of the NLRP3 blockade being a therapeutic target. Mridha A. attempted to block the NLRP3 protein complex in two murine models of steatohepatitis. They demonstrated that cholesterol crystals can activate the NLRP3 inflammasome in TLR-4/Myd88-primed macrophages in vitro and in vivo. Treatment with MCC950, a direct NLRP3 inhibitor in liver tissue, reduced the immune infiltration and mitigated liver injury and progression to fibrosis. Importantly, it was documented that the administration of MCC950 could not only suppress the development of fibrosis but reverse that which was already generated [45]. The latter could prove to change the course of the disease more widely, with a striking impact on the overall survival (OS) of patients and their quality of life and a substantial lightening of the burden of hospitalization and insurance expenses.

### 2.2. The Role of NLRP3 in Viral Hepatitis

More recent evidence proposes that the NLRP3 inflammasome pathway is implicated in the natural course of both HCV and HBV infections.

The non-CD81 uptake of HCV by Kuppfer cells comprises a stimulus for cellular PRRs. The HCV RNA induces the production of IL-1b through the TLR7 MyD88-dependent signaling pathway. In parallel, an K+ efflux triggers the activation of NLRP3 inflammasome for the cleavage and secretion of IL-1b, which further orchestrates the immune response [46]. Negash A. et al. demonstrated an alternative mechanism of NLRP3 activation in HCV. According to their research, the HCV core protein in hepatic macrophages triggers a calcium efflux interceded by phospholipase C [47]. IL-18, the other concomitant factor from NLRP3 activation, appears to stimulate the anti-viral effects of NK cells [48]. On the other hand, HBV, during its natural course, advances strategies to escape from the immune response. Related to this, HBeAg downregulates the NF-kB pathway and the generation of ROS, inhibiting the NLRP3 inflammasome. The above contributes to the HBV viral persistence [49].

**Figure 1:**
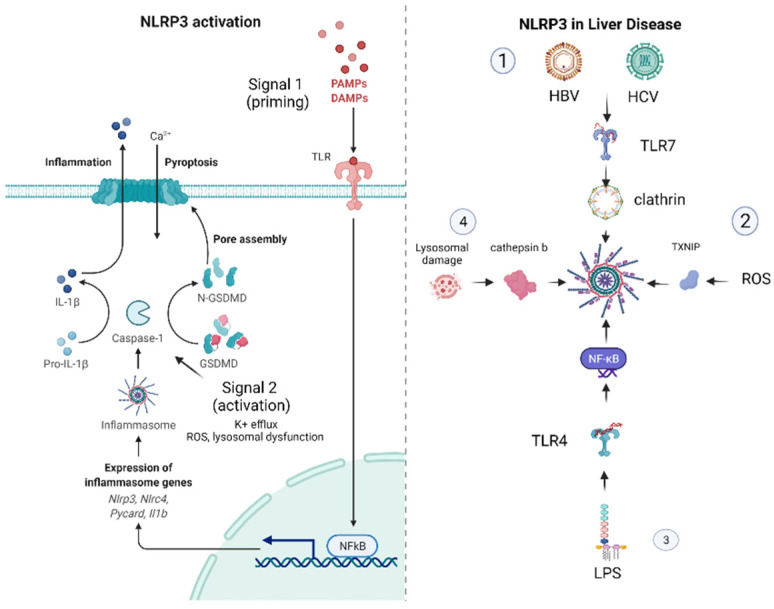
On the *left* is presented the activation of the NLRP3 inflammasome. PAMPs and DAMPs from the neighboring parenchyma stimulate the PRR system to upregulate the expression of NF-kB (Signal 1—priming), which triggers the expression of pro-ILs and the components of the inflammasome machinery. Additional signals from K+ efflux, ROS generation or lysosomal dysfunction activate the NLRP3 inflammasome. The activated caspase-1 potentiates the generation of active IL-1, IL-18 and gasdermin. The latter provokes pore formation in the cellular membrane, causing cell death and the release of inflammation mediators. On the *right* are reported the principal pathologic mechanisms in common liver diseases leading to NLRP3 activation: (1) HBV and HCV infection, (2) ARLD, (3) NASH and liver injury in sepsis and (4) the accumulation of lipid droplets.

A concise report about the role NLRP3 in liver disease (viral hepatitis, alcohol-related liver disease (ARLD), NASH and NAFLD) is presented in Figure 1.

## 3. The Role of NLRP3 in the Shaping of the HCC Tumor Microenvironment (TME)

As mentioned above, HCC develops in the vast majority of cases under the influence of chronic liver inflammation. Its microenvironment is comprised of a heterogeneous spectrum of cellular populations such as cancer-associated fibroblasts (CAFs) with the resultant extracellular matrix (ECM), HSCs, endothelial cells and various immunosuppressive aggregates. The latter are composed by an amalgam of myeloid-derived suppressor cells (MDSCs), tumor-associated macrophages (TAMs), tumor-associated neutrophils (TANs) and T regulatory cells (Tregs) [50]. In relapsed tumors, the microenvironment is characterized by the abundance of innate cells (e.g., dendritic cells and CD8+ lymphocytes with low toxicity) and diminished amounts of Tregs in comparison with primary tumors [51]. With the exception of MDSCs and Tregs, which consistently display a pro-tumorigenic phenotype exerting immune tolerance towards tumor cells, a sophisticated crosstalk within the tumor microenvironment (TME) polarizes the cellular populations in a context-specific manner, mediating tumor growth, invasion and metastasis as well as regulating angiogenesis and enforcing drug resistance [52]. It is of paramount importance to highlight the “double-edged sword” impact of inflammatory lytic programmed cell death during the initiation and progression of hepatocellular carcinogenesis. During the early stages of carcinogenesis, the immune response can repress tumorigenesis, potentiating tumor surveillance, e.g., IL-18 secretion activates natural killer (NK) cells to exert their cytotoxic potential against tumor cells; however, unprovoked continuous inflammasome activation and the presence of IL-1b in the TME attracts immunosuppressive cells such as MDSCs, which promote tumor invasion and metastasis [53]. These impacts are highlighted in a comprehensive study by Wei Q. et al., which investigated the expression of NLRP3 inflammasome in various stages of HCC [18].

Despite a recent surge in the literature of single cell analyses that demonstrate the existence of TAMs which synchronously express signatures from both the M1 and M2 macrophage clusters [54], TAMs and TANs are classically divided into two subtypes: on the one hand, M1 and N1 secrete IFN-γ and IL-12, potentiating the effector compartment of the cellular immunity, evading tumor growth; on the other hand, the M2 and N2 phenotypes, which are associated with the secretion of IL-4, IL-10 and TGF-β, induce an Th2 immunologic response [50,55,56]. The exact mechanisms by which TAMs, TANs and their contexts regulate HCC progression have been extensively reviewed elsewhere [50,56,57]. A continuum of transitions in the synthesis and composition of HCC TME can be observed based on integral causes such as the stochastic accumulation of mutations or extrinsic stimuli (e.g., chemotherapy). For example, the enhancement of Wnt/β-catenin signaling in cancer cells induces a c-myc-potentiated differentiation into M2 macrophages [58], while the administration of sorafenib augments the infiltration of TANs, which is accountable for the further recruitment of TAMs and Tregs [59]. The significance of metabolic reprogramming in the establishment of TME cannot be overstated enough. Aerobic glycolysis (or the Warburg effect), which is indispensable in order to provide building blocks to support the enhanced anabolic demands of proliferating tumor cells, generates substantial amounts of lactate. The presence of an acidic, nutrient-poor and hypoxic microenvironment results in the exhaustion of effector T-cells, polarizes the macrophages towards an M2 phenotype and impairs tumor-antigen presentation by dendritic cells [60]. Additionally, neutrophils are enforced to secrete neutrophil extracellular traps (NETs), promoting metastasis [61]. By virtue of intrinsic tumor hypoxia and drug-imposed hypoxia, and the fact that angiogenesis blocking constitutes a first- and second-line therapeutic choice in HCC, a shift towards fatty acid instead of glucose metabolism has been evidenced [62,63]. The importance of lipid metabolism was summarized exhaustively by Hu B. et al. [64]. It is of paramount importance to comprehensively investigate the role of exosomes as a means of HCC progression [52] and as a therapeutic moratorium [65], while the impact of ECM composition on the regulation of carcinogenesis is increasingly being inferred [66].

The importance of NLRP3 as a regulatory molecule in the pre-malignant stages of liver disease has been highlighted. It is well established that the NLRP3 inflammasome is released by pyroptotic hepatocytes and incorporated by adjoining cells, promoting inflammation and ECM deposition [67]. A growing body of recent literature has investigated the influence of the NLRP3 inflammasome on the configuration of the HCC microenvironment. Ding Y. et al. recently demonstrated that NLRP3 might orchestrate the infiltration of cellular populations in innate and adaptive immunity. In more detail, NLRP3 expression was associated with the degree of B cell, CD4+ T cell, CD8+ T cell, neutrophils, and dendritic cell (DC) invasion. This was reflected by the correlation of the immune, stromal and estimate score with NLRP3 expression. The above might be mediated by the mutation burden of the mismatch repair system (MMR) and co-expression with various methyl-transferases and immune checkpoint inhibitors such as lymphocyte-activation gene 3 (LAG3), inducible T cell co-stimulator (ICOS), cytotoxic T lymphocyte antigen 4 (CTLA4), T cell immunoglobulin, mucin domain-containing protein 3 (TIM3), programmed cell death protein 1 (PD1), programmed death-ligand 1 (PD-L1), PD-L2, T cell immunoglobulin and the ITIM domain (TIGIT) [68]. The latter appears to intercede the cytotoxic capacity of NK cells. NK cells carry several receptors on their surface, e.g., NKG2D. The abundance of their ligands in the TME is regulated by enzymes such as matrix metalloproteinases (MMPs), which regulate the composition of the ECM, among others. Lee H. et al. reported that the ablation of NLRP3 in HCC SK-Hep1 Luc cells resulted in elevated NK cytotoxicity in an IFN-g-independent mechanism. The involved mechanism constitutes the downregulation of MICA/B, a ligand of the activating NKG2D receptor, by the downregulation of MMP2, MMP9 and MMP14 in mice engrafted with NLRP3 KO(−/−) SK-Hep1 Luc cells [69]. Fatty acid oxidation (FAO) constitutes another process by which NLRP3 influences the HCC TME. The fibronectin type III structural domain-containing protein 5 (FNDC5) is frequently upregulated in HCC tissues, with a negative impact on the potential of tumor malignancy [70]. Liu H. et al. documented both in vitro and in vivo that FNDC5 induces the FAO-dependent M2 phenotype by blocking M1 polarization, downregulating the NF-κB/NLRP3 pathway [71]. Related to this, Zhang Q. et al. studied the contribution of FAO to the pro-inflammatory properties of an M2 subset in HCC, as they had previously documented the importance of the hypoxia-inducing factor (HIF)-1α/IL-1β signaling pathway [72]. They concluded that FAO-generated ROS drive, in an NLRP3-dependent manner, the expression of IL1-β, providing another potentially therapeutically targetable mechanism. In parallel, receptor-interacting protein kinase 3 (RIPK3), another regulator of fatty acid metabolism in TAMs, utilized the NLRP3 inflammasome machinery in order to become activated through caspase-1-mediated cleavage [73]. Finally, Tu C. et al. demonstrated that lactate and TGF-b exert their immunomodulatory effects in the TME partially by utilizing the NLRP3 inflammasome. Specifically, lactate can trigger inflammasome initiation on tumor-associated macrophages by building up ROS in parallel with tumor cell-derived TGF-b secretion. The latter exerts its function through the SMAD-autophagy-ROS signaling cascade [74]. It worth mentioning that evidence has begun to emerge about the regulatory role of the NLRP3 inflammasome in the tumor-stromal interconnection. Zan Y et al. demonstrated that the blockage of NEK7 in HCC cells could reduce the activation of HSCs [75]. As demonstrated above, the NLRP3 inflammasome, acting as a scavenging and effector system in the HCC TME, significantly shapes the anti-tumor immune response. Meanwhile, its effects in the tumor stroma requires further investigation.

## 4. The Role of the NLRP3 Inflammasome in the Therapeutic Management of HCC

The introduction of a wide variety of therapeutic approaches to the management of HCC has made the reformation of medical practice an experienced reality. Without exhaustive details, according to the latest European Society of Medical Oncology (ESMO) practice guidelines, patients who present with a single mass irrespectively of its size or with up to three nodules that are less than 3 cm each and have preserved liver function and Eastern Cooperative Oncology Group (ECOG) performance status (PS) are candidates for: hepatectomy, liver transplantation, thermal ablation or transarterial chemoembolization (TACE). Stereotactic body radiotherapy (SBRT), high dose rate (HDR)-brachytherapy and selective internal radiotherapy (SIRT) constitute alternative treatment approaches when management constrains arise. TACE comprises the standard of care in the treatment of patients presenting multinodular disease, ECOG PS 0 and efficient PS, with liver transplantation, resection, SIRT and systemic medical therapy being potent alternatives. Imaging-based signs of systemic disease, such as the invasion of portal veins or extrahepatic spread, render patients with preserved liver function and PS 0–2 as candidates for immunotherapeutic or anti-angiogenetic therapies with certain monoclonal antibodies and tyrosine kinase inhibitors (TKIs). Atezolizumab plus bevacizumab, and alternatively sorafenib or Lenvatinib, are considered the first line of treatment. Sorafenib, lenvatinib, cabozantinib, regorafenib and ramucirumab are authorized alternatives after the combination of atezolizumab and bevacizumab, while cabozantinib, regorafenib and ramucirumab constitute second line choices after sorafenib. For patients with end-stage liver disease, only supportive care measures are applied [76,77]. Concerning surgical techniques, an immense developmental evolution has radicalized surgical approaches. For minimal, unilobar or bilobar disease, “parenchyma-sparing liver surgery” constitutes an one-step resection. When the future liver remnant (FLR) is expected to be insufficient to sustain normal hepatic function after surgery, portal vein embolization (PVE) with the subsequent hypertrophy can be proposed as a treatment choice [78]. Two-stage hepatectomies (TSH)—i.e., conventional TSH with PVE only [79,80] and liver partition and portal vein ligation for staged hepatectomy (ALPPS) [81]—when performed in centers with sufficient experience, can offer significant survival advantages in patients with unresectable primary liver tumors [82,83].

### 4.1. The NLRP3 as Therapeutic Target

Given the above and taking into consideration a recent surge in clinical studies in the literature regarding the effects of the NLRP3 inflammasome system in HCC, it becomes evident that the NLRP3 inflammasome system could acquire a role in the therapeutic algorithm either as a therapeutic target or as a biomarker. In the first case, it could exert its usefulness as an adjuvant treatment in patients with low tumor burden or as part of systemic treatment in patients with greater tumor volume. The broad spectrum of NLRP3 activating stimuli, which was presented earlier, results in a diverse spectrum of signaling pathways and mechanisms that can influence HCC progression through NLRP3 activations such as ROS scavenging, immune cell reprogramming and metabolic reprogramming of the infiltrated immune cells. The vast majority the NLRP3 activations lead to tumor growth regression, with a few exceptions. The above is presented analytically in the Table 2.

### 4.2. The NLRP3 as Biomarker

Biomarkers are invaluable tools in our efforts to implement a more personalized approach in medical practice. Their application extends from HCC diagnosis, staging and tumor grading to guiding therapeutic management. Several studies [9] considering the NLRP3 as a biomarker in several malignancies have begun to accumulate [29]. The reduced expression of NLRP3 in colorectal cancer (CRC) tissue holds a positive predictive value [93]. Likewise, the upregulation of NLRP3 expression in breast cancer heralds a poorer five-year survival rate [94]. The capability of the NLRP3 inflammasome to promote an immune response provides the potential to predict the response to immunotherapy. In melanoma, the mutational burden of the *NLRP3* gene could provide useful data about the response to immunotherapy [95]. Unfortunately, despite the necessity of detecting and implementing into clinical practice new biomarkers for HCC [77], the evidence regarding the role of NLRP3 as a biomarker in HCC is limited. Wei Q. et al. investigated the expression of NLRP3 inflammasome components in accordance with HCC progression. They reported the inhibition of the expression of the inflammasome in HCC tissues, which was inversely correlated with the pathological grading and the HCC clinical stage [18]. Wang J. et al. developed a pyroptosis-associated computational algorithm to assess patient prognosis. The *NLRP3* gene had the highest mutation incidence. Copy number amplification was detected while its expression was significantly differentiated among HCC and the neighboring healthy tissue. Concerning the predictive value of NLRP3, the highest expression of NLRP3 was associated with poor OS [96].

## 5. Conclusions—Future Perspectives

Inflammation comprises a cardinal processes in liver homeostasis and disease [34]. Its significance has been studied conscientiously in the context of carcinogenesis [10], and immunotherapy with atezolizumab and bevacizumab constitutes the first line of therapy in patients with unresectable disease [97].

**Figure 2:**
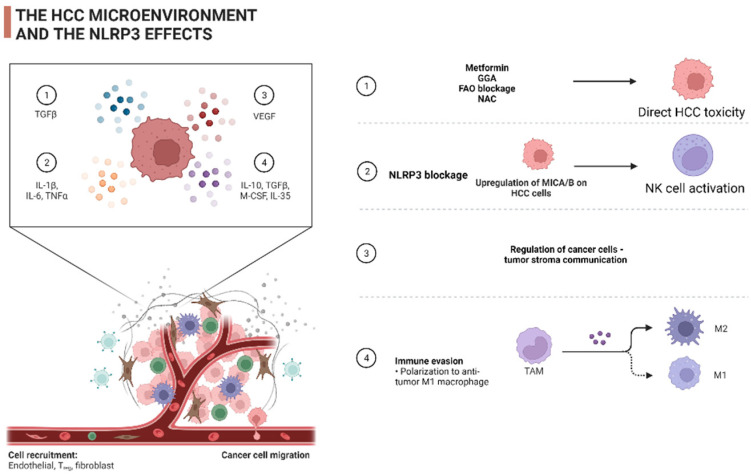
A brief outline of the therapeutic effects of the NLRP3 system in HCC.

The NLRP3 inflammasome appears to orchestrate the immune responses in liver diseases. It can be concluded that the activation of the NLRP3 inflammasome drives the progression from NAFLD to NASH, while hepatotropic viruses (e.g., HBV, HCV) downregulate it in order to evade immune invasion. Several candidate therapeutic targets that mediate the progression of NASH have been recognized, but clinical trials about their effects in humans are lacking. The generation of drugs that target the NLRP3 inflammasome would be game-changing in the management of NAFLD-driven HCC. On the other hand, the NLRP3 inflammasome appears to be an ideal therapeutic target in HCC. The NLRP3 inflammasome and its constituents are downregulated in HCC. This inhibition correlates with a greater disease stage and poorer differentiation [18]. Multiple mechanisms of HCC cytotoxicity by the therapeutic instrumentation of NLRP3 have been documented, such as direct hepatotoxicity, M1 polarization of TAM, the enhancement of NK cell activity and the regulation of the tumor–stromal communication. The pre-clinical data are encouraging, and their efficacy should be validated in clinical trials in the near future. The above therapeutic mechanisms are summarized in Figure 2.

Useful conclusions regarding role of the NLRP3 inflammasome in HCC carcinogenesis could be arrived at by analyzing the effects of other inflammasomes in different tissues. For example, the NLRP6 inflammasome constitutes a fundamental sensor of the intestinal mucosa and its downregulation is linked with alterations in gut microbiota [98] and a predisposition to inflammatory bowel disease and gastrointestinal cancer [24]. The latter is exerted by its assistance in the regulation of mucus secretion in goblet cells, where caspase-1 is required to activate the microtubule-associated protein 1A/1B-light chain 3 (LC3) and trigger mucus secretion [99]. Chen G. et al. demonstrated that *Nlrp6-knockdown* mice produce excessive amounts of inflammation and fail to heal damaged mucosa. The latter, in conjunction with the upregulation of TNF-a and IL-6, which stimulate the NF-kB and STAT3 pathways, promotes carcinogenesis [24]. A “steatohepatic subtype” has been recognized in HCC with alteration in the IL6/JAK-STAT signaling axis, and mutations in this pathway are frequent in HCC [6]. It would not be illogical to hypothesize that the downregulation of the NLRP3 inflammasome in HCC could potentiate carcinogenesis by the subsequent upregulation of tumor-promoting cytokines.

The role of the NLRP3 inflammasome is poorly investigated, and our scientific efforts should be pointed in this direction. A growing body of work in the literature substantiates that, in the years to come, the manipulation of the NLRP3 sensor pathway could prevent the progression to carcinogenesis in several patient groups.

## Figures and Tables

**Table 1:** Summarizes several cellular processes that can be therapeutically targeted.

Drug/Therapeutic Target	Study/Year/Reference	Study Subjects	Pathway	Outcomes
Obeticholic acid	Huang S. (2021) [38]	BMDM cells, hepatocytes/DIO + CCl4 mice	Inhibition of NLRP3 inflammasome activation in macrophages	Reduction in steatosis, fibrosis and immune infiltration
			Inhibition of lipid-induced NLRP3 inflammasome activation in hepatocytes	
Antcin A	Ruan S. (2021) [39]	KC cells/NAFLD mice	Inhibition of NLRP3 inflammasome activation in vitro/in vitro	Inhibition of immune infiltration
Auranofin	Hwangbo H. (2020) [40]	High-fat diet (HFD) NAFLD model	Inhibition of NLRP3 inflammasome, NOX4 and PPARγ activation	Inhibition of immune infiltration
Cardiolipin inhibitors (shRNA-CLS1)	Liu J. (2019) [41]	KC cells/methionine choline-deficient (MCD) diet mice	Inhibition of NLRP3 inflammasome activation in vitro/in vitro	Improvement in liver biochemistry
Cathepsin B inhibition	Tang Y. (2018) [42]	KC cells/MCD diet NASH mice model	Inhibition of NLRP3 inflammasome activation	Inhibition of immune infiltration and steatosis
Polyunsaturated fatty acid (PUFA)	Sui Y. (2016) [43]	HFD NASH mice model	Inhibition of NLRP3 inflammasome activation in vitro and in vivo	
Melatonin	Yu Y. (2021) [44]	db/m mice, db/db mice	Improvement in mitochondrial membrane potential (MMP)	Reduction in steatosis, fibrosis and immune infiltration
			Inhibition in NLRP3 inflammasome activation	

**Table 2:** A brief summary of the preclinical data with respect to NLRP3 inflammasome.

Drug/Therapeutic Target	Study/Year/Reference	Study Subjects	Pathway/Mechanism	Outcomes
Alpinumisoflavone (AIF)	Zhang Y. (2020) [84]	SMMC 7721, Huh7 cells	NLRP3-mediated pyroptosis	Reduction of tumor growth and metastatic potential
NEK7 inhibition	Yan Z. (2022) [75]	MHCC97L, HepG2 cell/mice	NLRP3-mediated pyroptosis	Reduction of tumor growth and metastatic potential
				Promotion of cancer cell-stromal communication
Biejiajian pills (BJJ)	Feng M. (2020) [85]	Diethyl nitrosamine-mediated hepatocarcinogenesis in SD rats	Dose-dependent reduction in NLRP3 activation	Reduction of tumor growth
Luteoloside	Fan S. (2014) [86]	Hep3B, SNU-449, Huh-7, MHCC- LM3 and MHCC97-H cell lines/BALB/c-nu/nu male mice	Downregulation of NLRP3 activation	Reduction of tumor growth and metastatic potential in vitro and in vivo
Metformin	Shen Z. (2021) [87]	BALB/c nude male mice	FOXO3-dependent induction of the NLRP3 inflammasome and autophagy	Reduction of tumor growth
Geranylgeranoic acid (GGA)	Yabuta S. (2020) [88]	HuH-7 cells	TLR4-induced ROS generation activating both non-canonical and canonical phases of pyroptosis	Reduction of tumor growth
NLRP3 siRNA or CPT1A blockage or N-acetyl cysteine (NAC) or etomoxir	Zhang Q. (2018) [89]	HepG2, Hep3B cells	Reduction in NLRP3 activation by FAO-mediated ROS	Reduction of HCC metastatic potential
17β-estradiol (E2)	Wei Q. (2015) [90]	BEL7402, SMMC7721 and HepG2 cells	ERβ/MAPK/ERK-mediated activation of NLRP3 inflammasome	Reduction of tumor growth
17β-estradiol (E2)	Wei Q. (2019) [91]	HepG2 cells	Autophagy reduction through E2/ERβ/AMPK/mTOR-induced NLRP3 activation	Reduction of tumor growth
IRAK1 blockage	Chen W. (2020) [92]	Huh7, Hep3B cells	Downregulation of NLRP3 activation through ERK/JNK pathway	Reduction of tumor growth
PPARγ inhibitors or FNDC5 blockage	Liu H. (2021) [71]	HepG2, SMCC7721 cells overexpressing FNDC5	Activation of the NF-κB/NLRP3 pathway	M1 TAM polarization
NLRP3 blockage	Lee H. (2021) [69]	HCC SK-Hep1 Luc, NK-92 cells	Upregulation of MICA/B on the HCC cells induced by NK activation through NKG2D receptor	Reduction in tumor growth and metastasis
RIPK3 mimic or FAO blockage	Wu L. (2020) [73]	Human HCC tissues	Activation of the ROS–Caspase1–PPAR pathway reversed M2 programming	Reduction in tumor growth
